# Advances in post-processing methods for multiple sequence alignment

**DOI:** 10.3389/fgene.2025.1722317

**Published:** 2025-11-10

**Authors:** Yixiao Zhai, Zitong Zhang, Zhen Li

**Affiliations:** 1 Institute of Fundamental and Frontier Sciences, University of Electronic Science and Technology of China, Chengdu, China; 2 School of Artificial Intelligence, Shenzhen University of Information Technology, Shenzhen, China; 3 Faculty of Computing, Harbin Institute of Technology, Harbin, China

**Keywords:** post-processing, meta-alignment, realigner, realignment, multiple sequence alignment

## Abstract

The reliability of multiple sequence alignment (MSA) results directly determines the credibility of the conclusions drawn from biological research. However, MSA is inherently an NP-hard problem, making it theoretically impossible to guarantee a globally optimal solution. Consequently, in addition to developing more efficient alignment algorithms, improving the quality of initial alignments through post-processing optimization has become an important strategy for enhancing the overall alignment accuracy. Although post-processing methods have shown potential in improving alignment accuracy, currently, there is a lack of systematic reviews and summaries. In this review, we provide a systematic overview of the development and key research ideas of MSA post-processing methods over the past 3 decades and outline potential directions for future research.

## 1 Introduction

Multiple sequence alignment (MSA) is a fundamental technique in bioinformatics. Its primary objective is to compare and align multiple biological sequences—such as DNA, RNA, or proteins—to reveal similarities and differences between them (Chao et al., 2022). The resulting alignments provide valuable insights into sequence homology and evolutionary relationships (Wang et al., 2024), and they also facilitate the identification of functional elements, conserved domains, and gene family members. These applications form the basis for a deeper understanding of the complexity and diversity of living systems. Therefore, the accuracy of MSA results is crucial for all downstream biological analyses.

Despite substantial progress, the quality of MSA results remains limited because of both intrinsic and extrinsic factors. On the objective side, the explosive growth of sequencing data (Wei Y. et al., 2022; Zhang et al., 2024), coupled with extensive sequence variability and experimental errors—such as base-calling inaccuracies and insertion–deletion biases—greatly increases the complexity of alignment and reduces its overall robustness (Chen et al., 2023; Edgar, 2022). On the algorithmic side, MSA is inherently an NP-hard problem; as a result, most existing tools rely on heuristic strategies that balance efficiency and accuracy, often at the expense of achieving a truly global optimum. Furthermore, current algorithms still face challenges in effectively modeling structural features and evolutionary divergence, which further constrains alignment precision.

Against this backdrop, post-processing methods for MSA have gained growing attention. The practical goal of these approaches is to directly enhance alignment accuracy and improve the reliability of downstream analyses. At the same time, the underlying algorithms of certain tools offer valuable insights and novel directions for advancing MSA technology. As biological data continue to expand in both scale and complexity, research in this area has become essential for improving the efficiency and robustness of bioinformatics analyses, thereby fostering scientific discovery and technological innovation in the field. In this review, we summarize the current MSA post-processing methods, highlighting their core principles, existing challenges, and potential future developments.

## 2 Methods

Traditional MSA tools typically rely on heuristic algorithms based on the principle of “once a gap, always a gap.” In other words, once an incorrect gap is introduced early in the alignment process, it tends to persist and continue to degrade the overall alignment quality. Various post-processing methods have been developed to address this issue, aiming to further enhance the accuracy and reliability of alignment results.

Currently, mainstream post-processing strategies for MSA can be classified into two categories:

1. Meta-alignment methods, which integrate multiple independent MSA results to produce more consistent and accurate alignments.
2. Realigner methods, which refine existing alignments by locally adjusting or re-evaluating regions with potential insertion or mismatch errors.

### 2.1 Meta-alignment

Meta-alignment tools take multiple MSA results, which are typically generated from the same unaligned sequence dataset using different alignment programs or parameter settings, as the input. The core idea is to fuse and optimize these initial alignments, integrating their respective strengths to construct a more consistent and accurate combined alignment. The resulting alignment not only preserves key information from each input result but may also reveal novel alignment patterns that are not captured by any single tool. The following section introduces several representative meta-alignment tools and their distinguishing features.

ComAlign (Bucka-Lassen et al., 1999) is one of the earliest meta-alignment methods proposed. Its core idea is that different MSA tools tend to produce distinct errors or approximations across various regions of the alignment. ComAlign addresses this by integrating the best performing segments from multiple alignments to generate a more accurate and robust consensus alignment. This method is built upon an extended dynamic programming framework: in an alignment matrix defined by *m* input sequences and *n* initial alignments, the paths corresponding to each initial alignment are annotated. The algorithm then identifies intersections and regions of agreement among these paths and iteratively integrates high-scoring segments to progressively construct the final consensus alignment. However, this process involves complex path searches and combinations in a high-dimensional dynamic programming space, resulting in high computational and memory demands. The original study validated the method only on nucleic acid datasets containing relatively short sequences and small sequence sets, and ComAlign struggles to scale effectively as the sequence length or number increases. Furthermore, due to its early publication, the source code of ComAlign is no longer available.

M-Coffee (Wallace et al., 2006) is currently the most widely used meta-alignment method for aligning both nucleic acid and protein sequences. Starting from multiple initial alignments, the method first constructs a consistency library. In this step, M-Coffee matches all pairs of characters (bases or amino acids) in each initial alignment with corresponding character pairs in other alignments. These character pairs are then weighted according to their consistency across the different alignments, thereby strengthening pairing signals that are supported by most initial alignments. Next, M-Coffee invokes the T-Coffee algorithm (Notredame et al., 2000) to generate the final MSA based on the consensus library. T-Coffee evaluates pairwise alignments to maximize the overall support of the matching character pairs within the library, thus producing a global alignment that best reflects the consensus among various alignment tools. However, this inclusive strategy also has potential drawbacks: if incorrect alignments are common across multiple initial results, they may be assigned higher weights as well. Consequently, M-Coffee’s overall accuracy depends strongly on the quality of its input alignments—typically approximating the average quality of the initial alignments and rarely surpassing the best among them.

AQUA (Muller et al., 2010) is a tool that encapsulates the meta-alignment workflow. Its input consists of the original, unaligned protein sequences. AQUA first automatically invokes MUSCLE3 (Edgar, 2004) and MAFFT (Katoh and Standley, 2013) to generate two initial alignments. It then employs the realigner RASCAL to refine these alignments, producing two corresponding realigned versions. Finally, the meta-alignment stage selects the most accurate alignment among the four candidate alignments (the two initial alignments and their realignments) based on the NorMD score (Thompson et al., 2001). As AQUA determines the best alignment from the outputs of multiple aligners, it can be regarded as a meta-alignment method. However, because the tool encapsulates the entire meta-alignment process, users cannot customize the initial inputs, and the range of candidate alignments is limited, which constrains its flexibility and adaptability to a certain extent.

MergeAlign (Colling et al., 2012) is designed to integrate multiple initial protein alignments. The method first represents these alignments as a weighted directed acyclic graph (DAG), in which nodes correspond to combinations of column positions and edges denote transitions between adjacent columns. Each edge is weighted by the number of initial alignments that contain the corresponding transition. The algorithm then identifies the path with the highest cumulative weight, and the nodes along this path form the merged alignment. Similar to M-Coffee, consensus regions supported by most initial alignments receive higher weights, enabling the final alignment to be synthesized from the collective input information. However, if alignment errors are commonly present across multiple initial results, they too may receive higher weights, potentially reducing the accuracy of the final alignment.

TPMA (Zhai et al., 2024a) is a state-of-the-art meta-alignment tool capable of integrating any number of nucleic acid MSAs. The method first ranks the input alignments in a descending order of their sum-of-pairs (SP) scores and then integrates them sequentially. Using a two-pointer algorithm, TPMA divides the two initial alignments into blocks containing identical sequence segments and merges those with higher SP scores into the final alignment. Owing to its simple algorithmic design and low computational and memory requirements, TPMA performs efficiently on large datasets. However, its performance remains highly dependent on the quality of the input alignments, and its relatively simple objective function imposes certain limitations in specific scenarios.

### 2.2 Realigner

Another category of the post-processing method for MSAs is to construct a realigner. Operating as standalone modules, realigners directly optimize and refine existing alignments without the need to re-run the entire alignment process. This approach can substantially improve the alignment accuracy while maintaining computational efficiency. Depending on the partitioning strategy adopted during the initial alignment, realigners are generally classified into three types: horizontal partitioning, vertical partitioning, and hybrid partitioning.

#### 2.2.1 Horizontal partitioning

**FIGURE 1:**
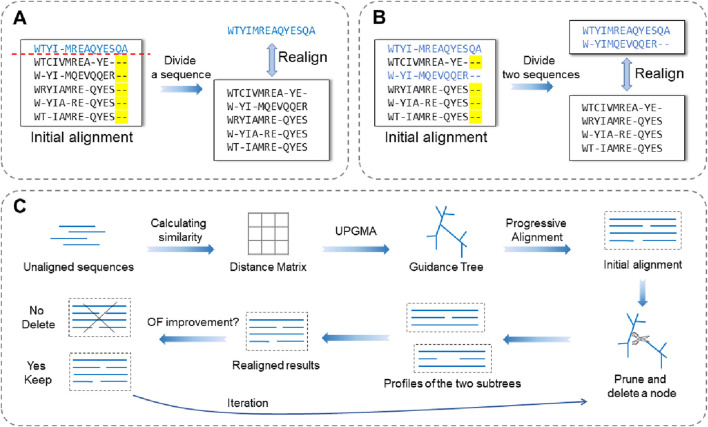
Schematic illustration of the three partitioning strategies used in horizontal partitioning methods. **(A)** Single-type partitioning. **(B)** Double-type partitioning, and **(C)** Tree-dependent partitioning.

A realigner that adopts a horizontal partitioning strategy typically operates through an iterative optimization process: in each iteration, the input alignment set is divided into two parts, which are then realigned to improve the alignment accuracy of the local area. Horizontal partitioning methods generally fall into three restrictive categories: single-type partitioning, double-type partitioning, and tree-dependent partitioning (Figure 1).

1. Single-type partitioning: one sequence is extracted from the initial alignment, whereas the remaining sequences form a profile. After removing the gaps from the extracted sequence, it is realigned against the profile in a sequence-to-profile manner.
2. Double-type partitioning: two sequences are extracted from the initial alignment to form one profile, whereas the remaining sequences form another. The two profiles are then aligned to each other in a profile-to-profile manner.
3. Tree-dependent partitioning: the initial alignment is divided into two subtree profiles based on the guide tree, and the profile-to-profile alignment is performed between the two subtrees.

The following section describes several typical realigners that use horizontal partitioning strategies.

ReAligner (Anson and Myers, 1997) is one of the earlier realigner tools, and it was primarily designed for DNA and RNA sequence data. It adopts a single-type partitioning strategy, in which each sequence is iteratively traversed and realigned. If a realignment improves the quality of the current alignment, the updated result replaces the original and serves as the input for the next iteration. This process continues until the alignment scores converge or stabilize.

The remove first (RF) method (Wallace and Higgins, 2005) is a realigner developed specifically for protein data and also employs a single-type partitioning strategy. Its iterative process and termination criteria are essentially the same as those of ReAligner. The main difference is that the RF method optimizes only one sequence per iteration, whereas ReAligner traverses all sequences in each iteration. In addition, its study also evaluated and compared double-type and tree-dependent partitioning strategies.

REFINER (Chakrabarti et al., 2006) is another realigner designed for protein sequences that use the single-type partitioning strategy. Its iterative process and termination criteria are similar to those of the RF method, but its optimization objective is more specific: REFINER aligns sequences to a family block model that represents conserved sequence or structural regions. Moreover, in each iteration, REFINER randomly selects a sequence and aligns it to a position-specific score matrix (PSSM) (Jones, 1999) generated from the remaining sequences rather than to a general profile.

ReformAlign (Lyras and Metzler, 2014) is a realigner for DNA and RNA data that does not follow any of the three partitioning strategies. The method first constructs a summarized profile based on the initial alignment. During each iteration, all sequences are realigned to this profile. If gaps are introduced in the process, the profile is fine-tuned accordingly, and all sequences are then realigned to the updated version. The iteration process continues until the alignment results remain unchanged for two consecutive rounds or a predefined maximum number of iterations is reached.

TreeRefiner (Manohar and Batzoglou, 2005) is another realigner designed for DNA and RNA data. Unlike the methods described above, it does not rely on the three partitioning strategies or an iterative optimization process. Instead, it performs realignment directly using a three-dimensional dynamic programming algorithm.

#### 2.2.2 Vertical partitioning

Realigners based on the vertical partitioning strategy are a relatively recent development, emerging within the past 5 years, later than tools using horizontal partitioning. These methods divide an initial alignment into contiguous blocks according to alignment columns and perform realignment within each block, thereby specifically correcting local low-quality regions and improving the overall alignment accuracy. Representative tools include Refin-Align, SpliVert, RPfam, and ReAlign-P, all of which are designed for protein sequences data.

**FIGURE 2:**
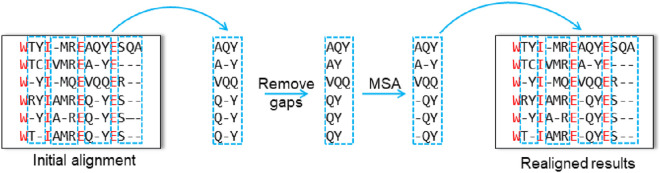
Schematic diagram of vertical partitioning method.

Refin-Align (Mokaddem et al., 2019) is the first realigner to use the vertical partitioning strategy, and it operates through an iterative optimization process. In each iteration, the method first divides the alignment into blocks according to columns that share identical amino acid compositions in the initial alignment. It then removes all gaps within these blocks and realigns each one using the Promalign (Mokaddem et al., 2018) tool. The program calculates the SP score for both the original and updated versions of each block; if the new block achieves a higher score, it replaces the original (Figure 2). Once all the blocks have been updated, the next iteration begins. This process continues until the results converge or a predefined iteration limit is reached.

SpliVert (Zhan et al., 2020) does not use an iterative optimization strategy but instead performs a single vertical partitioning realignment. This method first divides the initial alignment into three sections along the column axis: the head, middle, and tail. The authors note that protein sequences often exhibit complex structural characteristics, with the middle region generally being more conserved and the terminal regions being more variable. Based on this observation, SpliVert realigns only the middle region to minimize the influence of unstable flanking regions on the overall alignment, thereby enhancing the alignment quality. Specifically, the method removes gaps within the middle segment, realigns this portion independently, and then concatenates it with the original head and tail to generate the final optimized alignment.

RPfam (Wei et al., 2022b) uses a simulated annealing algorithm as its optimization strategy. In each iteration, the algorithm scans the current alignment to identify regions of low alignment quality and calculates their corresponding scores. It then randomly selects one of these low-quality blocks, locates the most poorly aligned segment within it, and realigns that segment using dynamic programming. If the new alignment outperforms the original, it replaces the previous version in the current alignment. The iterative process continues until the annealing temperature gradually decreases to a predefined threshold.

ReAlign-P (Zhai et al., 2025a), inspired by SpliVert, also focuses on realigning only the middle region of the alignment. Its key innovation lies in introducing a vertical iteration strategy. In each iteration, the algorithm first removes all gaps from the current alignment and then realigns the sequences using MAFFT. By comparing the new and original alignments, it identifies regions of complete identity and regions with discrepancies. Identical regions are preserved without further modification, whereas for discrepant regions, the SP score is calculated and the higher-scoring blocks are retained for the next iteration. The iteration terminates when the current alignment and the realignment result are completely identical.

#### 2.2.3 Hybrid partitioning

Hybrid partitioning combines horizontal and vertical partitioning techniques to synergistically improve the consistency and overall accuracy of alignment results by optimizing both the sequence and column levels.

RASCAL (Thompson et al., 2003) is the first realigner to adopt the hybrid partitioning strategy specifically aimed at re-optimizing protein MSAs. The method first uses the Secator tool (Wicker et al., 2001) to horizontally divide the initial alignment into sequence subfamilies. It then applies the NorMD objective function to compute the average column distance scores, thereby vertically identifying “core blocks”—regions that exhibit consistent and reliable alignments across most sequences. RASCAL constructs statistical models for these reliable regions and uses them to detect low-quality segments, which are subsequently realigned with a ClustalW-like algorithm (Larkin et al., 2007) to enhance the overall alignment quality.

Crumble and Prune (Roskin et al., 2011) is a realigner applicable to various types of biological sequences, and it uses a hybrid partitioning strategy to enhance both alignment efficiency and accuracy. The approach consists of two independent yet collaborative modules: Crumble, a vertical partitioning module, which addresses long sequence alignments by dividing them into shorter sub-problems, thereby reducing computational complexity, and Prune, a horizontal partitioning module, which targets large-scale alignments by splitting them into smaller subsets to improve computational efficiency. These two modules can be integrated within a job-tree framework, allowing the method to optimize the overall performance when processing complex, long, and deep alignments.

ReAlign-N (Zhai et al., 2024b) is a realigner designed for multiple nucleic acid sequence alignments, and it uses a hybrid partitioning strategy. The method consists of two modules: global realignment and local realignment. The global module uses a horizontal partitioning strategy, incorporating K-band technology and innovative memory-optimization schemes within a dynamic programming framework. In each iteration, all sequences are aligned against a summarized sequence profile, similar to that used by ReformAlign. The local module adopts a vertical partitioning strategy, identifying low-quality regions through exact match detection and entropy scoring, and it subsequently realigns and corrects these regions using MAFFT.

ReAlign-Star (Zhai et al., 2025b) is a realigner developed specifically for the star alignment tool (Tang et al., 2022; Zhou et al., 2024) and currently supports only nucleic acid sequences. The method uses a hybrid partitioning strategy. In the horizontal partitioning phase, a filtering mechanism identifies and removes low-quality “junk sequences” from the initial alignment while also eliminating gaps within these sequences for subsequent processing. During the vertical partitioning phase, a partial realignment is performed on the profile that excludes the low-quality sequences. Finally, the previously removed “junk sequences” are reintegrated with the updated profile using a sequence-to-profile approach to produce the final alignment.

## 3 Future direction

Several MSA tools already incorporate built-in iterative refinement mechanisms to enhance the alignment quality after the initial alignment stage. However, the improvements achieved by these mechanisms are often limited, particularly when the initial alignment contains systematic biases or when the input sequences differ substantially, resulting in suboptimal optimization outcomes. In contrast, dedicated post-processing approaches offer a more flexible and scalable framework for improving alignment accuracy. Nonetheless, compared with the extensive development of MSA algorithms themselves, research on post-processing methods remains relatively underexplored. Future efforts in this area could pursue breakthroughs in the following directions:

1. Efficient post-processing algorithms for ultra-large-scale datasets.

With the rapid advancement of technologies such as single-cell sequencing and metagenomics, the volume of biological sequence data is increasing exponentially. In this context, traditional MSA tools often struggle to deliver reliable results within practical time constraints, whereas newer tools tend to compromise the accuracy in favor of computational efficiency. This highlights an urgent need for post-processing algorithms capable of handling ultra-large-scale datasets. Existing post-processing tools still offer considerable room for improvement in computational efficiency and memory management. In particular, when aligning whole-genome alignments comprising millions of sequences, computational resources remain a critical bottleneck. Future research can explore the integration of high-performance computing and parallelization technologies, such as distributed computing frameworks, GPU acceleration, and emerging hardware architectures, to further reduce time and memory consumption, thereby enabling efficient alignment of datasets at the million-sequence scale and beyond.

2. Explore the extended application of vertical and hybrid partitioning strategies.

Future realigner designs are likely to prioritize vertical or hybrid partitioning strategies, as iterative optimization based solely on horizontal partitioning has shown limited potential for substantial improvement. At present, vertical partitioning approaches are primarily applied to protein sequence alignments, and their effectiveness for other data types, such as RNA sequences, coding regions, or noncoding regions, has yet to be systematically evaluated. Given the inherent advantage of vertical partitioning in refining local regions, future research could focus on extending these methods to genomic-scale datasets and developing specialized optimization algorithms tailored to specific functional regions.

3. Intelligent post-processing frameworks that integrate deep learning and pretrained models.

Current post-processing algorithms for MSA remain largely grounded in traditional heuristics and dynamic programming frameworks, with limited integration of deep learning or large-scale pretrained biological language models. In recent years, such models have shown remarkable potential in tasks including sequence feature extraction, structure prediction, and functional annotation. Integrating deep learning techniques with MSA post-processing algorithms could enable a deeper understanding of latent sequence features, paving the way for significant advances in alignment accuracy, robustness, and generalization.

## 4 Discussion

The accuracy of MSAs plays a decisive role in determining the reliability of downstream bioinformatics analyses. Post-processing methods have gained increasing research interest in recent years as a complementary approach to improving the alignment quality. In this review, we have systematically traced the evolution of MSA post-processing tools over the past 3 decades, providing a comprehensive overview of their conceptual foundations and methodological advances. Existing approaches can be broadly categorized into two major classes—meta-alignment and realigner methods—each with distinct design philosophies and optimization strategies. Finally, we have discussed the emerging challenges and future research directions in this field, highlighting opportunities for innovation at the intersection of algorithm design, large-scale data processing, and intelligent computational frameworks.
